# Pluripotent epigenetic regulator OBP-801 maintains filtering blebs in glaucoma filtration surgery model

**DOI:** 10.1038/s41598-020-77811-7

**Published:** 2020-12-01

**Authors:** Yuji Yamamoto, Atsushi Mukai, Toru Ikushima, Yasuo Urata, Shigeru Kinoshita, Junji Hamuro, Morio Ueno, Chie Sotozono

**Affiliations:** 1 Department of Ophthalmology, Kyoto Prefectural University of Medicine, 465 Kajii-cho, Hirokoji-agaru, Kawaramachi-dori, Kamigyo-ku, Kyoto, 602-8566 Japan; 2 Department of Frontier Medical Science and Technology for Ophthalmology, Kyoto Prefectural University of Medicine, Kyoto, 602-8566 Japan; 3 Oncolys BioPharma, Inc., Tokyo, 106-0032 Japan

**Keywords:** Glaucoma, Experimental models of disease, Drug development, Molecular medicine

## Abstract

Inhibition of fibrosis is indispensable for maintaining filtering blebs after glaucoma filtration surgery (GFS). The purpose of this study was to investigate the ability of a pluripotent epigenetic regulator OBP-801 (OBP) to ameliorate extracellular matrix formation in a rabbit model of GFS. Rabbits that underwent GFS were treated with OBP. The gene expression profiles and intraocular pressure (IOP) were monitored until 30 postoperative days. The bleb tissues were evaluated for tissue fibrosis at 30 postoperative days. In in vitro models, OBP interfered the functions of diverse genes during the wound-healing process. In in vivo GFS models, the expressions of *TGF-β*3, *MMP-2*, *TIMP-2* and *3*, *LOX, COL1A* and *SERPINH1* were significantly inhibited at 30 postoperative days in the OBP group compared with those in the vehicle control group. OBP treatment involving subconjunctival injection or eye drops showed no adverse effects, and reduced levels of α-SMA and collagen deposition at the surgical wound site. OBP maintained the long-lived bleb without scar formation, and IOP was lower at 30 postoperative days compared with the vehicle control group. These findings suggest that OBP is an effective and useful candidate low-molecular-weight agent for improving wound healing and surgical outcomes in a rabbit model of GFS.

## Introduction

It has been reported that wound healing is orchestrated by a differentiation process mediated by altered gene expression, i.e., a physiological response to the injured tissue^1^, which is associated with tissue fibrosis^2^. Thus, a more comprehensive understanding of these shared fibrosis pathways may ultimately lead to the development of effective and useful anti-fibrotic therapies. Fibrosis, the scarring that occurs in response to tissue injury, is defined as an excessive and persistent accumulation of extracellular matrix (ECM) components^3^, and can ultimately lead to organ dysfunction. The comprehensive analysis of a large amount of genes, regarding to these sequential processes, were performed. The genes were classified into six groups, namely the genes belonging to growth factors, ECM components, remodeling enzymes and mediators involved in cellular signaling, epithelial-to-mesenchymal transition and myofibroblast transformation (Supplementary Table 1). Accumulating evidence shows that epigenetic remodeling, including post-translational histone modifications, plays a role in the pathogenesis of fibrosis/tissue scarring through pro-fibrotic gene transcription^4^. In a previous study by Mann et al.^1^, the authors reported that with the design of specific epigenetic drugs, it may become possible to optimize therapeutic wound healing and prevent fibrosis in cases of chronic disease. However, there are still no effective anti-fibrotic therapies for reversing, stopping, or delaying the formation of scar tissue in most fibrotic organs^5^.

It is well-known that histone deacetylase inhibitors (HDACi) potently inhibit excessive fibrosis^6^. The anti-fibrotic action of HDACi is expected to become clinically applied for the treatment of lung, kidney, and heart disease^7,8^. From the point of view of wound healing following tissue injury, it was previously reported that HDACi inhibited corneal opacity after photorefractive keratectomy in a rabbit model^9^. Furthermore, it has been reported that in a rabbit model of GFS, the use of suberoylanilide hydroxamic acid (SAHA) had an anti-fibrotic effect on conjunctival tissue post-surgery, and that SAHA was actually safer than MMC for the protection of conjunctival tissue, potentially improving the surgical outcome^10^. However, the observation period in that study was short, being completed before scarring during the remodeling phase^11^. In addition, the dosage applied in that study might have been too high for practical application in a clinical setting.

It is widely known that surgical intervention is the primary treatment for glaucoma patients who are refractory to less-invasive conservative therapies. Although glaucoma filtration surgery (GFS) remains the 'gold standard', there are marked GFS-related problems that have yet to be resolved^12–14^. For example, the excessive amount of tissue scarring that occurs post-GFS has the tendency to induce the development of thick connective tissue around scleral flaps and filtration blebs, making it difficult to regulate and maintain a reduction of the patient's intraocular pressure (IOP) for a prolonged period post-surgery^15–17^, even by anti-proliferative agents such as 5-fluorouracil (5-FU) and mitomycin C (MMC)^18–22^, occasionally detrimental to corneal and conjunctival tissues^23–32^.

OBP-801 (OBP), a novel pluripotent epigenetic repressor of diverse genes that are involved in multiple steps of tissue inflammation and wound healing, as introduced in the present study, showed potent HDACi activity at a very low concentration, such as 1 nM, at levels that are 1/1000 times lower than the classical levels so far reported for HDACi, SAHA, valproic acid (VPA), and trichostatin A (TSA)^10,33^.

The aims of the present study were: to investigate the capacity of OBP to act as HDACi compared with SAHA, and examine the utility of this new HDACi to maintain filtering blebs without scar formation in a rabbit model of GFS, in order to develop a new practically applicable GFS method using an agent other than 5-FU or MMC.

## Results

### Inhibition of myofibroblast transition and LOX family expression

**Figure 1:**
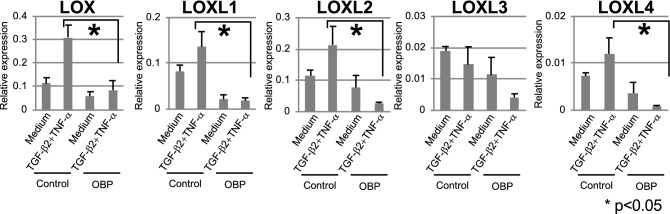
Effects of OBP on gene expression of *LOX* and *LOX* families (*LOXL1-4*) HConFs were pretreated with OBP (1 nM) for 24 h and then stimulated with 20 ng/mL TGF-β2 + 10 ng/mL TNF-α for 24 h. Gene expressions of *LOX* and *LOX* families (*LOXL1-4*) were measured by qRT-PCR. Threshold cycles (Ct values) were normalized to their corresponding GAPDH mRNA, and comparative mRNA levels were determined by the 2^(−Delta Ct)^ method. Error bars indicate the mean ± SD (n = 3). *P < 0.05.

To investigate the effect of OBP on fibrosis of human primary conjunctival fibroblasts (HconF), induction of myofibrosis by TGF-β2 or TGF-β2 + TNF-α was performed. HConF elicited a low level of background α-smooth muscle actin (α-SMA) expression detected by immunocytochemistry. Although TGF-β2 or TGF-β2 + TNF-α induced a significant transition of HconF into myofibroblasts, indexed by the increased expression of α-SMA, OBP almost completely inhibited these effects of TGF-β2 or TGF-β2 + TNF-α at a concentration of 1 nM (Supplementary Fig. 1). The lysyl oxidase (LOX) family is an important class of ECM crosslinking enzymes^34^. Of note, filtration blebs that play a pivotal role in maintaining a low IOP in GFS have been reported to show increases in the area and survival rate by the inhibition of LOXs^35^. Therefore, we also investigated the suppressive effect of OBP on the expression of LOX families in HConF. Expressions of *LOX*, *LOXL1*, *LOXL2*, and *LOXL4* were elevated by TGF-β2 + TNF-α stimulation, and these elevations were inhibited by OBP (Fig. 1).

### Inhibition of collagen production in HConF

**Figure 2:**
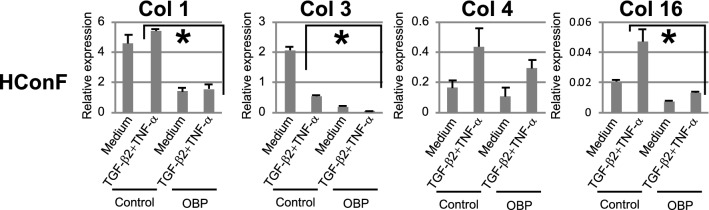
Effects of OBP on collagens (type 1, 3, 4, and 16) expressions. HConFs were pretreated with OBP (1 nM) for 24 h and then stimulated with 20 ng/mL TGF-β2 + 10 ng/mL TNF-α for 24 h. Gene expressions of collagens (type 1, 3, 4, and 16) were measured by qRT-PCR. Threshold cycles (Ct values) were normalized to their corresponding GAPDH mRNA, and comparative mRNA levels were determined by the 2^(−Delta Ct)^ method. Error bars indicate the mean ± SD (n = 3). *P < 0.05.

The elevated expressions of type 1, 3 and 16 collagens induced by TGF-β2 + TNF-α stimulation were suppressed in HConF by OBP (Fig. 2). The expression of type 1 collagen was induced by TGF-β2 + TNF-α, CTGF, PDGF-AA, and PDGF-CC (Supplementary Fig. 2a), type 4 collagen by TGF-β2 + TNF-α and PDGF-AA (Supplementary Fig. 2b), and type 16 collagen by TGF-β2 + TNF-α, CTGF, prostaglandin E2 and F2α, PDGF-AA, PDGF-BB, and PDGF-CC (Supplementary Fig. 2c). All of these elevated productions of collagen families by diverse fibrosis-inducing agents except type 1 collagen by PDGF-CC and type 16 collagen by prostaglandin F2α were suppressed by OBP.

### Inhibition of in vitro myofibroblast transition induced by TGF-β2 + TNF-α

**Figure 3:**
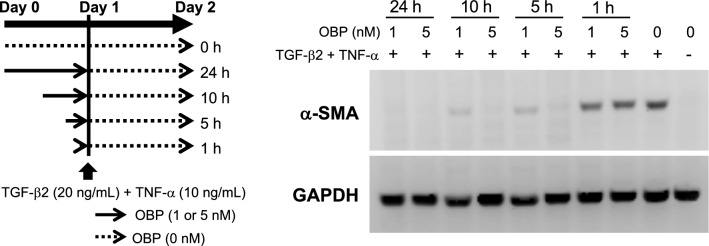
Effects of pulse treatment of HConFs by OBP on α-SMA expression. HConFs were pretreated with OBP (1 or 5 nM) for the indicated hours. It was washed out and then cells were stimulated with 20 ng/mL TGF-β2 + 10 ng/mL TNF-α for 48 h. The expression levels of each sample were evaluated by Western blot analysis.

The addition of OBP 24 h prior to TGF-β2 + TNF-α stimulation significantly suppressed the expression of α-SMA both at 1 and 5 nM OBP; however, when the addition was delayed to 5 or 10 h prior to the stimulation, significant inhibition was confirmed only at a concentration of 5 nM OBP. Only 1-h pulse of OBP before stimulation led to no suppression (Fig. 3).

### Myofibrosis was inhibited at an OBP concentration as low as 1 nM

A previous report described the inhibitory effect of a well-known HDACi, SAHA, on HConF myofibrosis. HconF fibrosis induced by TGF-β2 + TNF-α was inhibited by SAHA at a concentration of 250–500 nM, while OBP showed the same level of inhibition at a concentration of 0.5–1.0 nM. Immunocytochemistry and Western blot analysis showed that expressions of both type I collagen and α-SMA were inhibited with either 1 nM OBP or 500 nM of SAHA at the comparable level (Supplementary Fig. 3).

### Genes activated during the progression of tissue inflammation

**Figure 4:**
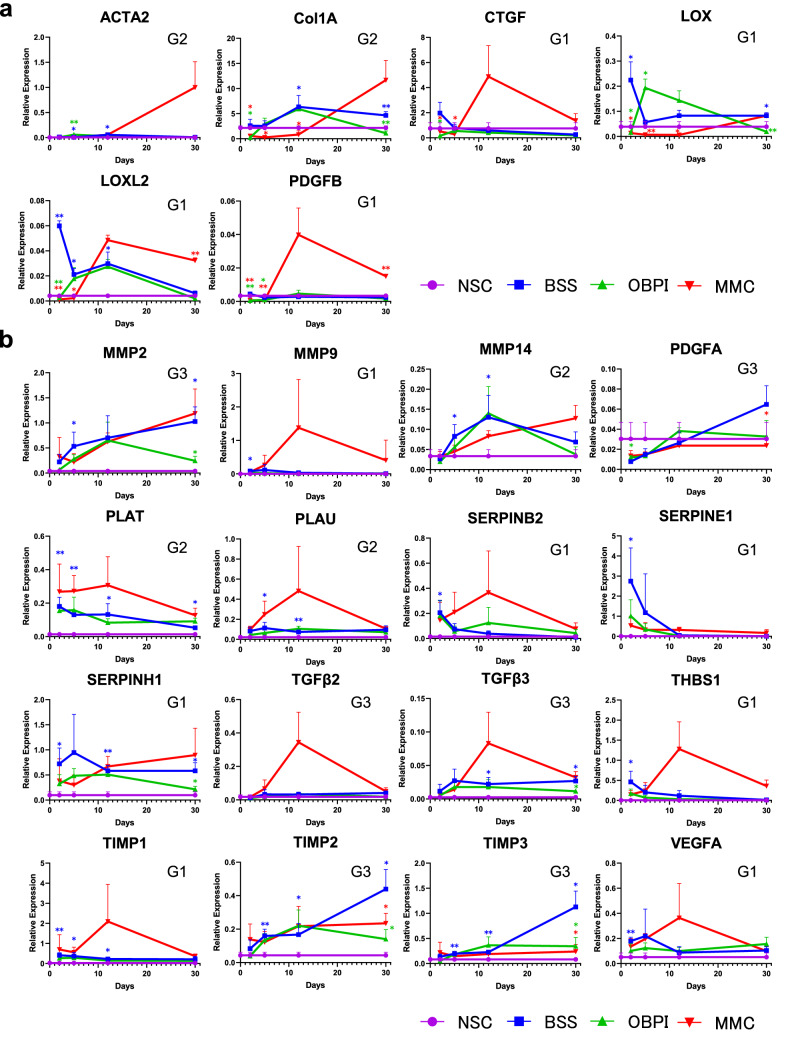
Time-course of fibrosis-related gene expression in bleb tissue of BSS Group, OBPI Group, and MMC Group at 2, 5, 12, and 30 days post-GFS. Non-surgical conjunctival tissue was used as a control (NSC). (**a**) Analysis by qRT-PCR. (**b**) Analysis by PCR Array. The peak elevation of fibrosis-associated gene expression in the Group-1 (G1) genes was between days 2 and 5 in BSS Group. The peak elevation of expression in the Group-2 (G2) genes was between days 5 and 12 in BSS Group. The peak elevation of expression in the Group-3 (G3) genes was on day 30 in BSS Group. Expressions of genes associated with fibrosis were inhibited in the OBPI Group at 30 days after surgery compared with BSS and MMC Groups. The number of biological replicates of each time point of each group was 3 to 4. Comparisons were performed by the *t* test between NSC and BSS Group on days 2, 5, 12, or 30 after GFS, with differences considered significant at **P < 0.01 or *P < 0.05 (blue). Comparisons were also performed by the *t* test between the BSS and OBPI Groups and between the BSS and MMC Groups at each time point, with differences considered significant at **P < 0.01 or *P < 0.05 (green and red respectively).

Considering the orchestration by differentiation processes accompanying altered gene expression^1^ during tissue inflammation and wound healing, we kinetically monitored the alteration of in vivo gene expression in the GFS rabbit models. The bleb tissues were sectioned, and the conjunctival tissues of the contralateral eyes were used as non-GFS controls. In the control group with balanced salt solution (BSS) injection, diverse genes such as growth factors, angiogenic factors [i.e., platelet-derived growth factor (*PDGF*) and vascular endothelial growth factor (*VEGF*)], cytokines [i.e., transforming growth factor-beta (*TGF-β*) and interleukins (*ILs*)], and genes associated with wound healing, fibrosis, and tissue remodeling were distinctly activated post-surgery. The major genes that were selected are listed in Fig. 4.

Of interest, the expression of many genes showed upregulation after GFS. Such elevation was common for *VEGF-A*, *TGF -β3*, matrix metallopeptidase (*MMP*)*-2*, *-9*, and *-14*, tissue inhibitor of metalloproteinase (*TIMP*)-*1*, *-2*, and *-3*, lysyl oxidase (*LOX*), collagen type I alpha (*COL1A*), actin, alpha 2 (*ACTA2*), plasminogen activator, tissue (*PLAT*), plasminogen activator, urokinase (*PLAU*), serpin family B member 2 (*SERPINB2*), serpin family E member 1 (*SERPINE1*), serpin family H member 1 (*SERPINH1*), and thrombospondin-1 (*THBS1*). Kinetically, the earliest peak elevation of expression between 2 to 5 days post-GFS was detected for *VEGF-A*, *MMP-9*, *TIMP-1*, *LOX*, *LOX-*like 2 (*LOXL2*), *CTGF, PDGF-B, SERPINB2*, *E1*, and *H1*, and *THBS1* (Group 1 genes)*,* intermediate peak elevation between 5 to 12 days post-GFS was detected for *COL1A*, *ACTA2*, *PLAT*, and *PLAU* (Group 2 genes)*,* and the latest peak elevation at 30 days post-GFS was detected for *TGF-β2* and *-β3*, *MMP-2*, and *TIMP-2* and -*3* (Group 3 genes)*.*

The above-described gene expressions showed distinctive tendencies to be either up- or downregulated by MMC (Fig. 4). Peak elevations of the expressions of most Group 1 genes were upregulations except for *LOX*, *LOXL2*, *SERPINE1*, and *SERPINH1,* and they moved to day 12. *LOX* and *SERPINH1* moved to day 30. *SERPINE1* was fixed on day 2. Two Group 2 genes, *COL1A* and *ACTA2*, were markedly upregulated and moved to day 30. The peak elevations of *TGF-β2* and *-β3* in Group 3 were upregulations and they moved to day 12, whereas those of *TIMP-2* and *-3* were downregulations and fixed on day 30.

### OBP modulation of wound/scar-related gene expression

The OBP Group showed the inhibition of many genes associated with tissue inflammation and remodeling, as compared with those genes in both the BSS and MMC Groups (Fig. 4). Compared with the BSS Group, *TGF-β3*, *MMP-2*, *TIMP-2* and *-3*, *LOX*, *COL1A*, and *SERPINH1* were significantly inhibited in the OBPI Group at 30 days post-GFS.

**Figure 5:**
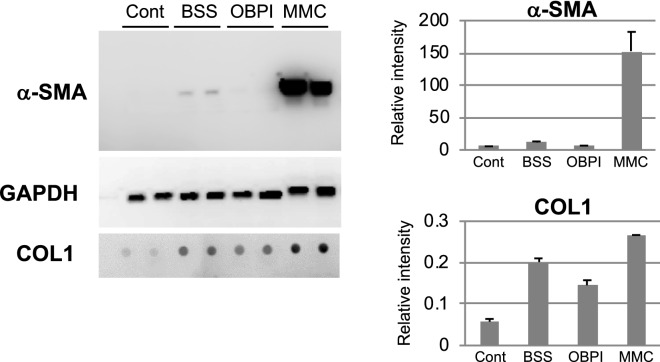
Expressions of α-SMA and collagen-1 in bleb tissues. Western blot analysis was performed at 30 days post-GFS in BSS, OBPI, and MMC Groups. Non-surgical conjunctival tissue was used as a control. OBP treatment reduced the expression levels of α-SMA and collagen-1 compared with BSS- or MMC-treated eyes. Error bars indicate the mean ± SD (n = 2).

At 12 days post-GFS, *ACTA2* and *THBS1* were also inhibited. The peak elevated gene expression of *LOXL2* was significantly inhibited at 2 days post-GFS. The *MMP-2* and *14* and *TIMP-1* genes played a critical role in tissue remodeling and elicited the opposing changes in MMC and OBP Groups (MMC: activation; OBP: inhibition), whereas *TIMP*-*2* and *-3* genes were inhibited in both groups at 30 days post-GFS (Fig. 4). Of interest, *COL1A* was elevated by MMC but significantly inhibited by OBP at 30 days post-GFS. *ACTA2* was also elevated by MMC at 30 days post-GFS, whereas this gene expression was elevated by OBP at 5 days post-GFS and down-regulated at 30 days post-GFS (Fig. 4). Western blot analysis was performed using rabbit conjunctival bleb tissue treated with BSS, OBP, and MMC at 30 postoperative days (Fig. 5). OBP treatment (subconjunctival injection; SI) reduced the expression levels of α-SMA and collagen-1 compared with BSS- or MMC-treated eyes.

### OBP enabled IOP lowering until 30 days after GFS

**Figure 6:**
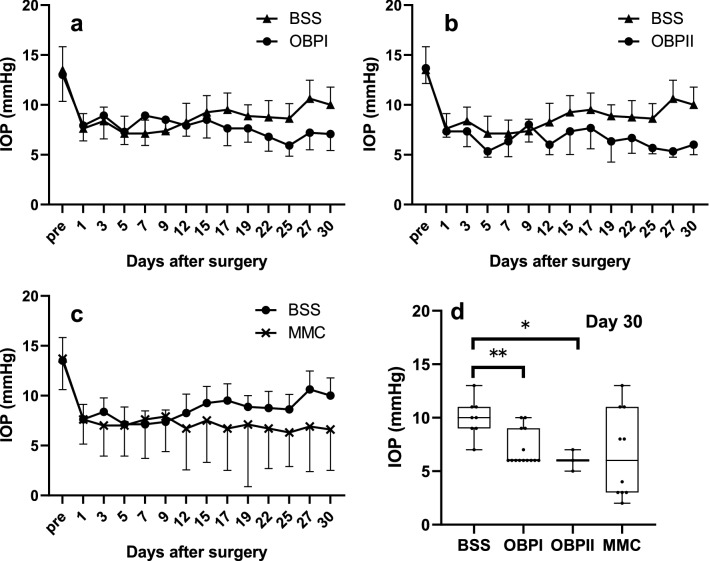
Postoperative IOP of rabbit GFS models. (**a**,**b**) IOP in rabbit eyes treated with OBP (either by SI or OI) remained significantly lower than that of rabbits treated with BSS between 15 to 30 days post-GFS. (**c**) The lowering of IOP in the eyes treated with MMC was not significant. (**d**) At 30 postoperative days (final test period), IOP in OBP-801-treated eyes remained significantly lower and in a stable range than that in BSS-treated eyes. In MMC-treated eyes, the mean IOP was low, yet it varied widely among the treated eyes. The box and whisker plot with individual data points demonstrates the median (line) as well as lower and upper interquartile range (IQR; box), whiskers show the highest and lowest values of IOP, with differences considered significant at **P < 0.01 or *P < 0.05. The numbers of rabbits of BSS, OBPI, OBPII and MMC were 8, 14, 3, and 10 respectively.

**Figure 7:**
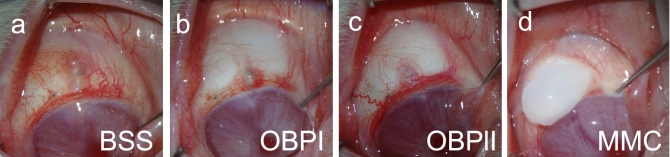
Macroscopic postoperative photographs of rabbit eyes on day 30 after surgery. (**a**) Treatment with BSS was associated with a flat bleb with hyperemia. (**b**,**c**) OBP-801 treated eyes showed a flat, functional bleb with a normal vascular appearance. (**d**) Treatment with MMC was associated with an avascular, thin, and cystic bleb, and the conjunctiva around the bleb was scarred, resulting in an encapsulated bleb.

Post-GFS, IOP in rabbits treated with BSS remained low for approximately 2 postoperative weeks, and then gradually increased (Fig. 6a–c). IOP in the OBP Groups (either by SI or ocular instillation; OI) remained lower than that in the BSS-Group rabbits, even from 2 weeks until 30 postoperative days (the final observation day) (Fig. 6a,b). IOP in the OBP-treated eyes was significantly lower than that in the BSS-treated eyes until 30 postoperative days [repeated measures design, BSS Group vs. OBPI Group (*P* < 0.0001, Fig. 6a), and BSS Group vs. OBPII Group (*P* = 0.017, Fig. 7b). On the other hand, IOP in the MMC-treated eyes was not significantly lower than that in the BSS-treated eyes until 30 postoperative days (*P* = 0.55, Fig. 6c). In 7 of 10 rabbits treated with MMC, hypotony occurred within 30 postoperative days (IOP < 5 mmHg). However, it did not occur in any of the rabbits treated with OBP (either by SI or OI) or BSS, except for in the period immediately after surgery. At 30 postoperative days, IOP in the OBP-treated eyes remained significantly lower in comparison with that in BSS-treated eyes [Wilcoxon test, BSS Group vs. OBPI Group (*P* = 0.0022, Fig. 6d), and BSS Group vs. OBPII Group (*P* = 0.023, Fig. 6d)] and within a stable range. In the MMC-treated eyes, the mean IOP was low, but it varied among treated eyes. Of note, hypotony was still observed in 5 of the 10 rabbits treated with MMC at 30 postoperative days. Moreover, an increase of IOP recurred in the eyes of some rabbits treated with MMC (Fig. 6d). At 30 postoperative days, IOP in the MMC-treated eyes was not significantly lower than that in BSS-treated eyes (*P* = 0.11, Fig. 6d).

### OBP induced unscarred blebs with normal vascular appearance

Blebs treated with BSS gradually became flat at around 3 postoperative days. Conjunctival hyperemia peaked at 3 postoperative days, and gradually disappeared at around 15 days after surgery. From 15 to 30 postoperative days, no major change in the appearance of bleb conjunctival tissue was observed. Treatment with BSS was associated with a flat, not avascular, and scarred bleb at 30 postoperative days (Fig. 7a, Supplementary Fig. 4a–c). The appearance of the OBP Group followed a similar course to that of the BSS Group. Treatment with OBP by SI or OI was associated with a flat, not avascular, and non-scarred bleb at 30 postoperative days (Fig. 7b,c). Conversely, the conjunctival blood vessels of the blebs treated with MMC began to disappear at 3 postoperative days and became completely avascular at 7 postoperative days. Scarring occurred around the filtration bleb and it became encapsulated at 3–5 postoperative days, initially forming a very large filtration bleb, but the bleb gradually became smaller until 30 postoperative days. Treatment with MMC was associated with thin-walled and cystic blebs, and the conjunctiva around the blebs was firmly scarred, resulting in the blebs becoming encapsulated (Fig. 7d, Supplementary Fig. 4j–l). Two of 10 rabbits treated with MMC developed a shallow anterior chamber (Data not shown). Conversely, no shallow anterior chamber was observed in rabbits treated with BSS or OBP. Moreover, irrespective of the treatment group, no rabbits showed leakage of aqueous humor, corneal edema, corneal opacity, endophthalmitis, nor cataract.

### OBP inhibited the expression of fibrous deposits

**Figure 8:**
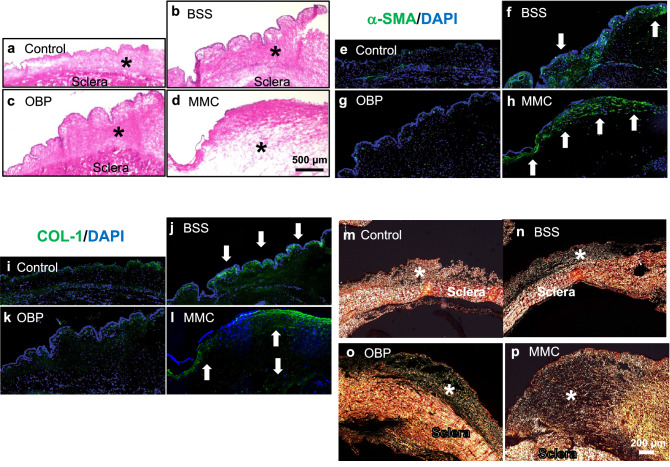
Microscopic analysis of bleb tissues of rabbit GFS models on day 30 after surgery. The images show representative sections from the blebs in each group. (**a**–**d**) Hematoxylin and eosin staining. (**e**–**l**) Immunofluorescence staining for α-SMA and collagen type 1 (COL-1). (**m**–**p**) Picrosirius red staining. OBP reduced expressions of α-SMA and COL-1, and decreased collagen deposition in bleb tissue after GFS, whereas expressions of α-SMA and COL-1 and collagen deposition were increased in MMC-treated rabbit bleb tissues. Scale bar: (**a**–**l**): 500 μm. (**m**–**p**): 200 μm. Asterisks show bleb tissues.

Hematoxylin and eosin (H&E) staining of bleb tissues treated with OBP via SI showed the mildest form of fibrous deposition in conjunctival tissue compared with tissues treated with BSS or MMC, and tissues treated with MMC showed the densest form of fibrous deposition in conjunctival tissue (Fig. 8a–d). Furthermore, OBP treatment via SI reduced the expression levels of α-SMA and collagen-1 immunostaining compared with BSS- or MMC-treated eyes. In contrast, and more than via BSS treatment, MMC treatment increased the expression levels of α-SMA and collagen-1 immunostaining (Fig. 8e–l). Picrosirius red staining revealed that the bleb tissues treated with OBP via SI contained the mildest form of collagen fibers compared with tissues treated with BSS or MMC, and those treated with MMC contained the densest form of yellow collagen fibers (Fig. 8m–p).

## Discussion

The primary aim of this study was to clarify the features of OBP to modulate the expression of wound/scar-related genes both in vitro and in vivo (Figs. 1, 2, 3, 4, 5), and the secondary aim was to investigate the effects of SI or OI of OBP on subconjunctival scarring, bleb maintenance, and reduction of IOP in in vivo GFS rabbit models, aiming to obtain information on the safety and tolerance of this HDACi agent for future application in a clinical setting (Figs. 6, 7, 8).

The LOX family is involved in ECM crosslinking^34^ and filtration blebs showed increases in area and survival rate by the inhibition of LOXs^35^. Interestingly, the elevated expressions of *LOX*, *LOXL1*, *LOXL2,* and *LOXL4* in HconF by TGF-β2 + TNF-α were inhibited by OBP (Fig. 1). Other classes of ECM relevant to the wound-healing process and participating in fibrosis involve collagen families. The elevated expressions of collagen 1, 3, and 16 in HconF were inhibited by OBP (Fig. 2). Furthermore, several isoforms of collagen families, elevated by diverse fibrosis-inducing mediators such as TGF-β2 + TNF-α, CTGF, PGE2, and PDGF, but not PGF2α, were also inhibited by OBP (Supplementary Fig. 2). Of note, all of these biological effects were reproducibly confirmed at a concentration as low as 1 nM. All of these observations prompted us to investigate the in vivo effect of this new HDACi.

The IOP of rabbit eyes treated with OBP remained significantly lower than that of eyes treated with BSS control, and no serious complications were observed. Conversely, some rabbits treated with MMC developed avascular blebs, hypotony with shallow anterior chambers, or a recurrence of an IOP increase nearly to the same level as the preoperative IOP within 30 postoperative days.

Conjunctival wound healing is a complex process, involving the proliferation of fibroblasts, transition of fibroblasts into activated myofibroblasts, ECM remodeling, contraction, and angiogenesis^36–38^. Although diverse anti-inflammatories and antimetabolites have been investigated, no agents have been reported to be as efficacious as antifibrotics including MMC and 5-FU, which have been widely used to reduce postoperative conjunctival fibrosis and scar formation^20,39–41^. However, these antifibrotics reportedly exhibit cytotoxic side effects detrimental to conjunctival tissue^23,24^, such as avascular and thin-walled filtration blebs^25–27^, hypotony^26,28,29^, and bleb infection^30–32^. As part of the continuing efforts to identify new candidate agents, some researchers have investigated the topical application of compounds during surgery^42^. Rho-associated protein kinase (ROCK) inhibitors (i.e., AMA0526 and Y-27632) reportedly improve the surgical outcome in a rabbit model of GFS^42,43^. It has also been reported that metalloprotease inhibitors modulate the postoperative scarring post-GFS^44^. Of note, the practicality of dosages is not simply due to drug concentration, but due to balance of drug efficacy and side effects. In this context it is the urgent issue to define this balance of OBP, compared with SAHA and many other candidate compounds now under development.

HDACi reportedly elicits antifibrotic effects in multiple organs, including the cornea^9,45–48^. Sharma et al.^10^ reported that SAHA prevented excessive wound healing and scar formation in a GFS rabbit model, resulting in a lowered IOP and decreased vascularity. In that animal study, the clinical outcomes were almost comparable to those of OBP, except for the difference of the nearly 1000-times lower dosage of OBP than SAHA. Importantly, conjunctival tissues from patients in whom GFS failed due to scarring showed a several-fold increase in type 1 collagen transcripts^49^.

The present study’s findings revealed that OBP inhibited expressions of diverse genes associated with fibrosis, including *TGF-β3*, *CTGF*, *PDGFA*, *PDGFB*, *LOX*, *LOXL2, ACTA2*, *COL1A2*, *SERPINH1*, *MMP-2*, and *TIMP-2 and 3* at various time-points (Fig. 4). An elevated expression of *COL1A* was evident at 30 days post-GFS on treatment with MMC*,* while OBP injection resulted in no elevated expression of the gene from 2 to 30 postoperative days (Fig. 4). Compared with the BSS Group, the OBP Group showed significant inhibition of *TGF-β3*, *MMP-2*, *TIMP-2* and *-3*, *LOX*, *COL1A*, and *SERPINH1* at 30 postoperative days. All of these observations were consistent with the previously reported finding that improvement of the surgical outcome by the inhibition of MMP is mediated through reduced scar tissue production^44^. Also, the previously observed elevated expression of TGF-β after GFS suggests that TGF-β2 and 3 play pivotal roles in scarring and wound contracture after GFS^50–52^.

Histologic evaluation and Western blot analysis revealed that in contrast to MMC, the expression levels of α-SMA and collagen-1 were not elevated in eyes treated with OBP compared with eyes treated with BSS at 30 postoperative days (Figs. 5, 8). During the follow-up period in this study, the filtration bleb became dysfunctional and IOP increased in eyes treated with BSS. Surprisingly, in eyes treated with MMC, the genes involved in fibrosis, i.e., *COL1A*, *ACTA2*, *CTGF*, *SERPINH1,* and *LOXL2*, were not inhibited, but rather promoted at 30 postoperative days. This may be biologically plausible, considering the cytotoxic effect of MMC resulting in tissue damage due to excessive fibrosis. Considering the delay and upregulation of the peak elevated expression of wound healing-related gene and the excessive deposition of fibrous proteins in the MMC-treated bleb tissues, we suggest that MMC lowers IOP after surgery by maintaining the injured tissue via the delay of wound healing. However, by delaying the wound healing, excessive fibrosis does occur secondarily. The remodeling caused by the excessive fibrosis may have induced the recurrence of increased IOP post-surgery. Moreover, a cytotoxin may have made the conjunctival wall of the blebs avascularized and thinned, resulting in high rates of late-onset bleb leaks and related infections.

Recently, Futakuchi et al.^53^ confirmed that SAHA induced the alteration of gene expression involved in the TGF-β receptor signaling pathway, cell proliferation, ECM organization, inflammatory responses, and angiogenesis. Our findings also showed that OBP markedly inhibited expression of the angiogenic cytokine PDGF, consistent with the findings in a previous in vivo study of SAHA, in which bleb vascularity was decreased^10^. In previous studies, anti-VEGF treatments were shown to improve the surgical success rate of GFS in a rabbit model by reducing bleb vascularity and collagen deposition^54,55^. Our findings suggest that OBP may support the maintenance of filtration blebs also via its antiangiogenic effects. For clinical application, the effect of long-time combination of OBP and steroid have to be examined in future.

In conclusion, the topical administration of OBP eye drops resulted in the maintenance of low-level IOP after GFS (Fig. 6c,d). OBP regulates diverse genes and interferes with the wound-healing process at different levels as a pluripotent epigenetic repressor. The dosages in this study, both for injection and application as eye drops, were in the range of around 10 ng or lower. Therefore, they were much lower than dosages of the agents thus-far reported. Our findings suggest that OBP may be a physiological agent that targets the wound-healing process to improve the outcome of GFS.

## Materials and methods

### Regents for cell culture

SAHA was purchased from Cayman Chemical (Ann Arbor, MI, USA). OBP was provided by Oncolys BioPharma Inc. (Tokyo, Japan). A 250 μM stock solution and 10 μM sub-stock solution of OBP and 10 mM stock solution of SAHA were prepared by dissolving them in dimethyl sulfoxide (DMSO; Miltenyi Biotec, Bergisch Gladbach, Germany) and storing them at − 30 °C.

### Cell culture

HconFs (ScienCell Research Laboratories, Carlsbad, CA, USA) were cultured in fibroblast medium supplemented with 10% fetal bovine serum (Thermo Fisher Scientific, Waltham, MA, USA) and antibiotics (penicillin [100 U/mL]/streptomycin [100 μg/mL] (WAKO, Osaka, Japan). HconFs at passages 2–4 were used in all experiments.

HconF cells were grown to 80–90% confluence, and the medium was replaced with serum-free medium containing SAHA (1 μM) or OBP (1 nM) from 24 h before the induction of fibrosis. Recombinant human TGF-β2 (20 ng/mL) and/or TNF-α (10 ng/mL), recombinant human PDGF-AA, BB, CC, and DD (100 ng/mL each) (R&D Systems, Abingdon, UK), prostaglandin E2, F2α (50 μM each), and human recombinant CTGF (500 ng/mL) (WAKO) were used to examine the effects of fibrosis.

### GFS rabbit model: agents and administration

A 250 μM stock solution and 10 μM sub-stock solution of OBP was prepared by dissolving it in DMSO, and it was subsequently stored at − 30 °C. OBP was then dissolved at 10 or 100 nM in BSS (Alcon, Fort Worth, TX, USA). According to our preliminary in vivo experiment and previous reports^10,53^, OBP at a concentration 10- or 100-times higher than that resulting in the in vitro suppression of α-SMA (*ACTA*) and collagen type 1 (*COL1A*) was administered by SI or OI, respectively.

The stock solution MMC (Kyowa Hakko Kirin Co., Ltd., Tokyo, Japan) was dissolved in BSS at 0.2%, and then diluted to 0.02% at the time of use. The route of BSS, OBP, and MMC administration was by either SI or OI, and the regimen applied was well-tolerated without serious adverse events. Rabbits undergoing GFS were classified into the following 4 treatment groups: (1) BSS Group (n = 20 rabbits that received a 200-μL SI of BSS at 30 min pre-GFS and at 1, 3, and 5 postoperative days); (2) OBPI Group (n = 26 rabbits that received a 200-μL SI of 10 nM OBP at 30 min pre-GFS and at 1, 3, and 5 postoperative days); (3) OBPII Group (n = 3 rabbits that received 4 eye drops of 20 μL of 100 nM OBP solution at 30-s intervals at 30 min pre-GFS and twice daily at 1 through 7 postoperative days); (4) MMC Group (n = 26 rabbits that received a 100-μL SI of 0.02% MMC solution at 3 min pre-GFS) (Table 1).

**Table 1:** Rabbit GFS model treatment groups.

Groups	Treatment	Concentration	Volume (μl)	Routes	Timing (days)							
					0	1	2	3	4	5	6	7
BSS	BSS		200	SI	◯	◯		◯		◯		
OBPI	OBP	10 nM	200	SI	◯	◯		◯		◯		
OBPll	OBP	100 nM	80	OI	◯	◯	◯	◯	◯	◯	◯	◯
MMC	MMC	0.02%	100	SI	◯							

Rabbits undergoing GFS were divided into 4 treatment-specific groups based on the types of drugs (BSS, OBP, or MMC). Only Group OBPII was treated by ocular instillation (OI). The other groups were treated by subconjunctival injection (SI).

*SI *subconjunctival injection, *OI *ocular instillation.

### GFS rabbit model

**Figure 9:**
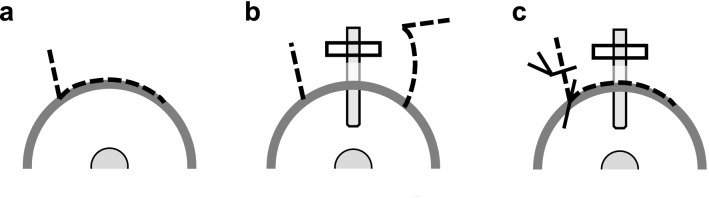
Schematic diagrams of the GFS method. (**a**) First, a fornix-based conjunctival flap is made. (**b**) A silicone cannula (22-gauge) is then inserted into the anterior chamber and fixed with 10-0 nylon suture. (**c**) The silicone cannula is then covered by the conjunctival flap, resulting in an aqueous-filtering bleb.

Adult female Japanese white rabbits (2.5–3.0 kg in weight) were used for the GFS rabbit-model experiments performed in this study. All procedures were conducted in accordance with the ARVO Statement for the Use of Animals in Ophthalmic and Vision Research, and the study protocols were approved by the Animal Use Committee of Kyoto Prefectural University of Medicine, Kyoto, Japan. For adaptation to the laboratory environment, all rabbits were kept in separate cages in the laboratory for one week prior to undergoing GFS. For the rabbit model of GFS, we chose a cannula model instead of a filtration-surgery model in order to maintain a permanent fistula to drain aqueous humor into the subconjunctival wound site (Fig. 9)^50,56^. On the day of the operation, GFS was performed on the right eye of each rabbit under general anesthesia with intramuscular injections of a mixture of 50-mg/kg ketamine hydrochloride (Daiichi Sankyo Propharma Co., Ltd., Tokyo, Japan) and 10-mg/kg xylazine hydrochloride (Bayer Yakuhin, Ltd., Osaka, Japan), combined with a topical administration of oxybuprocaine eye drops (Santen Pharmaceutical Co., Ltd., Osaka, Japan). The GFS method performed followed the previously reported technique^50,56^. First, a partial-thickness 6-0 nylon (Handaya Co., Ltd., Tokyo, Japan) corneal traction suture was placed in the superior cornea to more easily visualize the superior conjunctiva. Next, a fornix-based conjunctival flap was made to a distance of 15 mm from the limbus. Using a microvitreoretinal (MVR) blade (Mani, Inc., Tochigi, Japan), a partial-thickness scleral tunnel was then made from 4 mm behind the limbus to the anterior corneal stroma. A 22-gauge/25-mm intravenous cannula (Terumo Corporation, Tokyo, Japan) was then inserted through the scleral tunnel into the anterior chamber. Next, a cannula was inserted into the mid-pupillary area, with the needle then being withdrawn. The cannula was trimmed, and 10-0 nylon suture (Mani) was then used to fix the cannula to sclera. Finally, the conjunctival incision was closed with 10-0 nylon suture, and the bleb formation was tested (Fig. 9). At the end of surgery, 1 drop of 1% atropine sulfate (Nitten Pharmaceutical Co., Ltd., Nagoya, Japan) and ointment of 0.1% betamethasone sodium phosphate and 0.35% fradiomycin sulfate (Shionogi & Co., Ltd., Osaka, Japan) was applied. The ointment of 0.1% betamethasone sodium phosphate and 0.35% fradiomycin sulfate was also applied at 1, 2, 3, 5, and 7 postoperative days. Each surgery was performed by a single experienced surgeon. All evaluation of subjective/qualitative grading measures such as bleb appearance and histology of rabbits underwent GFS were carried out by different persons than the one who performed the GFS surgeries in a masked fashion.

### Reverse transcriptase-polymerase chain reaction (RT-PCR) analysis

In the analysis of bleb tissues, the BSS, OBPI, and MMC Group rabbits were first sacrificed with a lethal intravenous injection of pentobarbital (Kyoritsu Seiyaku Corporation, Tokyo, Japan) at 2, 5, 12, and 30 postoperative days. Both eyes were enucleated, and the bleb tissues were isolated. The contralateral eye of each rabbit was used as the non-surgical control. Total RNAs were isolated from the cultured cells and bleb tissues by the RNeasy Mini Kit (QIAGEN, Inc., Valencia, CA, USA). Immediately after, the RNA concentration and quality were measured with Agilent Bioanalyzer 2100 (Agilent Technologies, Santa Clara, CA, USA). The analyses were performed as described in supplementary information.

### Western blot analysis

For the analysis of cultured cells, cells were lysed with M-PER Mammalian Protein Extraction Reagent (Thermo Fisher Scientific) containing protease/phosphatase inhibitor cocktail (Thermo Fisher Scientific). The lysate was collected in a 1.5-mL tube and centrifuged at 12,000*g* for 10 min at 4 °C to pellet the cell debris. The supernatants were transferred to new tubes and the protein concentration was measured using the Pierce BCA protein assay kit (Pierce Biotechnology, Inc., Rockford, IL, USA). For the analysis of bleb tissues, each rabbit was sacrificed at 30 postoperative days and treated with procedures identical to those described above. The isolated bleb tissue was put into a 1.5-mL tube and frozen in liquid nitrogen. The frozen tissue was then ground in a chilled mortar, with the crushed tissue collected in a new 1.5-mL tube and lysed with radioimmunoprecipitation assay (RIPA) buffer containing protease/phosphatase inhibitor cocktail (Thermo Fisher Scientific). The tissue was then sonicated at 30-s intervals for 5 min and rotatory mixed for several hours. Next, the supernatant was collected by centrifugation at 10,000*g* for 20 min at 4 °C, and the protein concentration was measured using the Pierce BCA protein assay kit (Pierce Biotechnology, Inc.). The analyses were performed as described in supplementary information.

### Immunocytochemistry

Cells were fixed for 10 min at − 30 °C with cold methanol, and blocked with 1% FBS in PBS for 60 min at room temperature. The cells then were stained with the antibodies anti α-SMA (1/2000) (Sigma-Aldrich), anti-Type I (1/100), and IV (1/100) collagen (Abcam). As secondary antibodies, Alexa Fluor 594 Goat Anti-Mouse IgG (1/1000) and Alexa Fluor 488 Goat Anti-rabbit IgG (1/1000) (Thermo Fisher Scientific) were used. Cell nuclei were then stained with DAPI (5 μg/mL) (Dojindo, Kumamoto, Japan) and the cells were inspected with the fluorescence microscope BZ9000 (Keyence, Osaka, Japan).

### Clinical evaluation of the effects in GFS

To assess the adverse effects, clinical examinations were performed to evaluate the general and bleb appearance and IOP data were obtained prior to GFS and at 1, 3, 5, 7, 9, 12, 15, 17, 19, 22, 25, 27, and 30 postoperative days. IOP was measured in both eyes of each rabbit with a tonometer (ICARE TONOVET; Icare Finland Oy, Vantaa, Finland), and was performed three times per eye, with the median value of the three measurements then calculated^57^.

### Histologic evaluation

At 30 days post-GFS, each rabbit was sacrificed, both eyes were enucleated, and the bleb tissues were sectioned. Conjunctival tissue obtained from the non-operated contralateral eye of each rabbit was used as a control. The tissues were stained with H&E and picrosirius red stain using a Picrosirius Red Stain Kit (Polysciences, Inc., Warrington, PA, USA). Immunofluorescence staining for α-SMA, a marker of myofibroblasts, using mouse anti-actin α-smooth muscle antibody (Sigma-Aldrich) and collagen-1 using anti-collagen I antibody (Abcam) was performed. Images were obtained using a dual-sensor, charge-coupled device (CCD), microscope digital camera (DP80; Olympus Corporation, Tokyo, Japan), and a fluorescence microscope (BZ-9000; Keyence).

### Statistical analyses

In vitro data on mRNA expression were analyzed by the *t* test using Microsoft Excel (Microsoft, Seattle, WA, USA). In vivo data on mRNA expression were analyzed by the *t* test, and IOP data were analyzed by the repeated measure design and Wilcoxon test, using JMP version 13 statistical software (SAS Institute, Cary, NC, USA). A *P*-value of < 0.05 was considered significant.

## Supplementary information

Supplementary Information.

## References

[1] Mann J, Mann DA (2013). Epigenetic regulation of wound healing and fibrosis. Curr. Opin. Rheumatol..

[2] Zeisberg M, Kalluri R (2013). Cellular mechanisms of tissue fibrosis. 1. Common and organ-specific mechanisms associated with tissue fibrosis. Am. J. Physiol. Cell Physiol..

[3] Wynn TA, Ramalingam TR (2013). Mechanisms of fibrosis: therapeutic translation for fibrotic disease. Nat. Med..

[4] Yao HW, Li J (2015). Epigenetic modifications in fibrotic diseases: implications for pathogenesis and pharmacological targets. J. Pharmacol. Exp. Ther..

[5] Robinson CM, Watson CJ, Baugh JA (2012). Epigenetics within the matrix: a neo-regulator of fibrotic disease. Epigenetics.

[6] O'Reilly S (2017). Epigenetics in fibrosis. Mol. Aspects Med..

[7] Pang M, Zhuang S (2010). Histone deacetylase: a potential therapeutic target for fibrotic disorders. J. Pharmacol. Exp. Ther..

[8] Tang J, Yan H, Zhuang S (2013). Histone deacetylases as targets for treatment of multiple diseases. Clin. Sci..

[9] Sharma A, Mehan MM, Sinha S, Cowden JW, Mohan RR (2009). Trichostatin a inhibits corneal haze in vitro and in vivo. Investig. Ophthalmol. Vis. Sci..

[10] Sharma A (2016). Epigenetic modification prevents excessive wound healing and scar formation after glaucoma filtration surgery. Investig. Ophthalmol. Vis. Sci..

[11] Lockwood A, Brocchini S, Khaw PT (2013). New developments in the pharmacological modulation of wound healing after glaucoma filtration surgery. Curr. Opin. Pharmacol..

[12] Landers J, Martin K, Sarkies N, Bourne R, Watson P (2012). A twenty-year follow-up study of trabeculectomy: risk factors and outcomes. Ophthalmology.

[13] Kirwan JF (2013). Trabeculectomy in the 21st century: a multicenter analysis. Ophthalmology.

[14] Sugimoto Y (2015). Intraocular pressure outcomes and risk factors for failure in the collaborative bleb-related infection incidence and treatment study. Ophthalmology.

[15] Addicks EM, Quigley HA, Green WR, Robin AL (1983). Histologic characteristics of filtering blebs in glaucomatous eyes. Arch. Ophthalmol..

[16] Hitchings RA, Grierson I (1983). Clinico pathological correlation in eyes with failed fistulizing surgery. Trans. Ophthalmol. Soc. U. K..

[17] Schlunck G, Meyer-ter-Vehn T, Klink T, Grehn F (2016). Conjunctival fibrosis following filtering glaucoma surgery. Exp. Eye. Res..

[18] Lama PJ, Fechtner RD (2003). Antifibrotics and wound healing in glaucoma surgery. Surv. Ophthalmol..

[19] Wilkins M, Indar A, Wormald R (2005). Intra-operative mitomycin C for glaucoma surgery. Cochrane Database Syst. Rev..

[20] Seibold LK, Sherwood MB, Kahook MY (2012). Wound modulation after filtration surgery. Surv. Ophthalmol..

[21] Wong MH (2013). The Singapore 5-fluorouracil trial: intraocular pressure outcomes at 8 years. Ophthalmology.

[22] Cabourne E, Clarke JC, Schlottmann PG, Evans JR (2015). Mitomycin C versus 5-fluorouracil for wound healing in glaucoma surgery. Cochrane Database Syst. Rev..

[23] Shapiro MS, Thoft RA, Friend J, Parrish RK, Gressel MG (1985). 5-Fluorouracil toxicity to the ocular surface epithelium. Investig. Ophthalmol. Vis. Sci..

[24] Elner VM (2009). Aberrant wound-healing response in mitomycin C-treated leaking blebs: a histopathologic study. Arch. Ophthalmol..

[25] Palanca-Capistrano AM (2009). Long-term outcomes of intraoperative 5-fluorouracil versus intraoperative mitomycin C in primary trabeculectomy surgery. Ophthalmology.

[26] Membrey WL, Poinoosawmy DP, Bunce C, Hitchings RA (2000). Glaucoma surgery with or without adjunctive antiproliferatives in normal tension glaucoma: 1 intraocular pressure control and complications. Br. J. Ophthalmol..

[27] Anand N, Arora S, Clowes M (2006). Mitomycin C augmented glaucoma surgery: evolution of filtering bleb avascularity, transconjunctival oozing, and leaks. Br. J. Ophthalmol..

[28] Zacharia PT, Deppermann SR, Schuman JS (1993). Ocular hypotony after trabeculectomy with mitomycin C. Am. J. Ophthalmol..

[29] Kupin TH, Juzych MS, Shin DH, Khatana AK, Olivier MM (1995). Adjunctive mitomycin C in primary trabeculectomy in phakic eyes. Am. J. Ophthalmol..

[30] Jampel HD (2001). Risk factors for late-onset infection following glaucoma filtration surgery. Arch. Ophthalmol..

[31] Razeghinejad MR, Havens SJ, Katz LJ (2017). Trabeculectomy bleb-associated infections. Surv. Ophthalmol..

[32] Yamamoto T (2014). The 5-year incidence of bleb-related infection and its risk factors after filtering surgeries with adjunctive mitomycin C: collaborative bleb-related infection incidence and treatment study 2. Ophthalmology.

[33] Seet LF, Toh LZ, Finger SN, Chu SWL, Wong TT (2019). Valproic acid exerts specific cellular and molecular anti-inflammatory effects in post-operative conjunctiva. J. Mol. Med..

[34] Vallet SD, Ricard-Blum S (2019). Lysyl oxidases: from enzyme activity to extracellular matrix cross-links. Essays Biochem..

[35] Van Bergen T (2013). The role of LOX and LOXL2 in scar formation after glaucoma surgery. Investig. Ophthalmol. Vis. Sci..

[36] Chang L, Crowston JG, Cordeiro MF, Akbar AN, Khaw PT (2000). The role of the immune system in conjunctival wound healing after glaucoma surgery. Surv. Ophthalmol.

[37] Wynn TA (2008). Cellular and molecular mechanisms of fibrosis. J. Pathol..

[38] Klingberg F, Hinz B, White ES (2013). The myofibroblast matrix: implications for tissue repair and fibrosis. J. Pathol..

[39] Zada M, Pattamatta U, White A (2018). Modulation of fibroblasts in conjunctival wound healing. Ophthalmology.

[40] Georgoulas S, Dahlmann-Noor A, Brocchini S, Khaw PT (2008). Modulation of wound healing during and after glaucoma surgery. Prog. Brain Res..

[41] Hollo G (2017). Wound healing and glaucoma surgery: modulating the scarring process with conventional antimetabolites and new molecules. Dev. Ophthalmol..

[42] Van de Velde S (2015). Rho kinase inhibitor AMA0526 improves surgical outcome in a rabbit model of glaucoma filtration surgery. Prog. Brain Res..

[43] Honjo M (2007). Potential role of Rho-associated protein kinase inhibitor Y-27632 in glaucoma filtration surgery. Investig. Ophthalmol. Vis. Sci..

[44] Wong TT, Mead AL, Khaw PT (2005). Prolonged antiscarring effects of ilomastat and MMC after experimental glaucoma filtration surgery. Investig. Ophthalmol. Vis. Sci..

[45] Niki T (1999). A histone deacetylase inhibitor, trichostatin A, suppresses myofibroblastic differentiation of rat hepatic stellate cells in primary culture. Hepatology.

[46] Rombouts K (2002). Trichostatin A, a histone deacetylase inhibitor, suppresses collagen synthesis and prevents TGF-beta(1)-induced fibrogenesis in skin fibroblasts. Exp. Cell Res..

[47] Wang Z (2009). Suberoylanilide hydroxamic acid: a potential epigenetic therapeutic agent for lung fibrosis?. Eur. Respir. J..

[48] Kitano A (2010). Therapeutic potential of trichostatin A to control inflammatory and fibrogenic disorders of the ocular surface. Mol. Vis..

[49] Seet LF (2017). Upregulation of distinct collagen transcripts in post-surgery scar tissue: a study of conjunctival fibrosis. Dis. Models Mech..

[50] Mead AL, Wong TTL, Cordeiro MF, Anderson IK, Khaw PT (2003). Evaluation of anti-TGF-β2 antibody as a new postoperative anti-scarring agent in glaucoma surgery. Investig. Ophthalmol. Vis. Sci..

[51] Cordeiro MF, Bhattacharya SS, Schultz GS, Khaw PT (2000). TGF-beta1, -beta2, and -beta3 in vitro: biphasic effects on Tenon's fibroblast contraction, proliferation, and migration. Investig. Ophthalmol. Vis. Sci..

[52] Esson DW (2004). Expression of connective tissue growth factor after glaucoma filtration surgery in a rabbit model. Investig. Ophthalmol. Vis. Sci..

[53] Futakuchi A (2017). Molecular mechanisms underlying the filtration bleb-maintaining effects of suberoylanilide hydroxamic acid (SAHA). Investig. Ophthalmol. Vis. Sci..

[54] Li Z (2009). Inhibition of vascular endothelial growth factor reduces scar formation after glaucoma filtration surgery. Investig. Ophthalmol. Vis. Sci..

[55] Nilforushan N, Yadgari M, Kish SK, Nassiri N (2012). Subconjunctival bevacizumab versus mitomycin C adjunctive to trabeculectomy. Am. J. Ophthalmol..

[56] Cordeiro MF, Gay JA, Khaw PT (1999). Human anti-transforming growth factor-beta2 antibody: a new glaucoma anti-scarring agent. Investig. Ophthalmol. Vis. Sci..

[57] Ma D (2016). Repeatability, reproducibility and agreement of intraocular pressure measurement in rabbits by the TonoVet and Tono-Pen. Sci. Rep..

