# Supplementary material for: Pluripotent epigenetic regulator OBP-801 maintains filtering blebs in glaucoma filtration surgery model
Source: Sci Rep. 2020 Dec 1;10:20936. doi: 10.1038/s41598-020-77811-7 (PMC7708845; doi:10.1038/s41598-020-77811-7)
Supplement: Supplementary file 1 — Supplementary Information. [file 41598_2020_77811_MOESM1_ESM.pdf]

Pluripotent epigenetic regulator OBP-801 maintains filtering blebs in glaucoma filtration surgery model

Yuji Yamamoto,[1] Atsushi Mukai,[1] Toru Ikushima,[1] Yasuo Urata,[3] Shigeru Kinoshita,[2] Junji Hamuro,[1] Morio Ueno,[1] and Chie Sotozono[1]

[1] Department of Ophthalmology, Kyoto Prefectural University of Medicine, Kyoto, Japan.

[2] Department of Frontier Medical Science and Technology for Ophthalmology, Kyoto Prefectural University of Medicine, Kyoto, Japan.

[3] Oncolys BioPharma, Inc., Tokyo, Japan.

Supplemental Information

Supplementary Figure 1

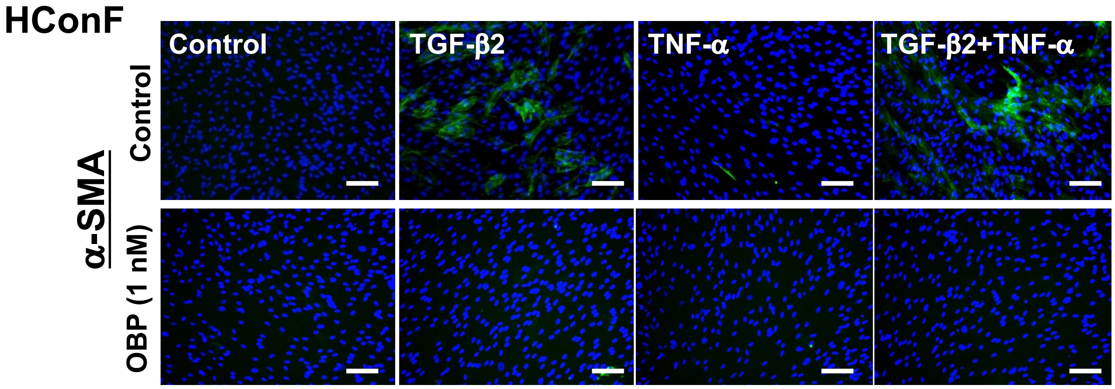

Supplementary Figure 1. Effects of OBP on α-SMA expression. HConFs were pretreated with OBP (1 nM) for 24 hours and were stimulated with 20 ng/mL TGF-β2, 10 ng/mL TNF-α and TGF-β2+TNF-α for 48 hours. The samples were evaluated by immunocytochemistry. TGF-β2 and TGF-β2+TNF-α induced α-SMA expression (green) was reduced by OBP pretreatment. Scale bar = 100 μm.

Supplementary Figure 2

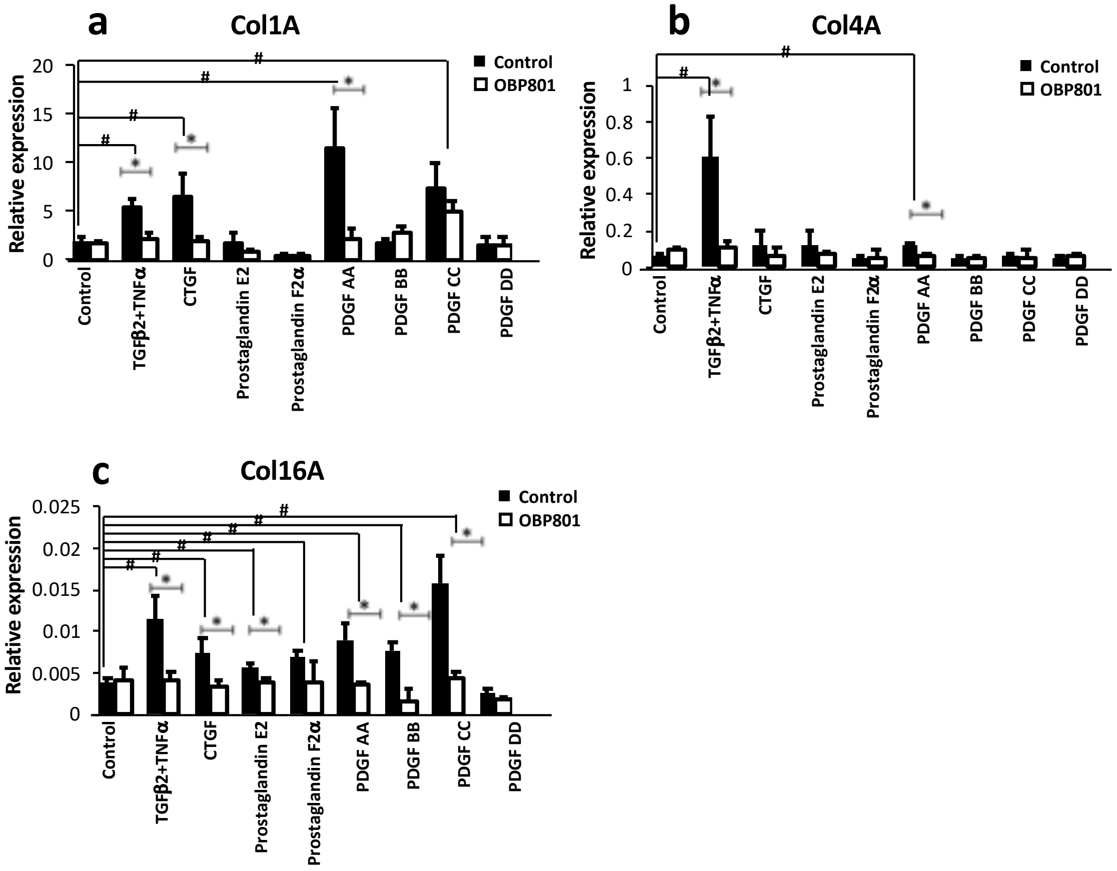

Supplementary Figure 2. Effects of OBP and various fibrosis inducers on collagens (type 1, 4, and 16) expression. HConFs were pretreated with OBP (1 nM) for 24 hours and were stimulated with indicated reagents for 24 hours respectively. mRNA levels of collagens were measured by quantitative RT-PCR. Threshold cycles (Ct values) were normalized to their corresponding GAPDH mRNA and the comparative mRNA levels determined by the 2^(-Delta Ct) method. Error bars indicate the mean ± SD (n = 3). # $P < 0.05$ for the difference between untreated and fibrosis inducers - treated HconFs. * $P < 0.05$ for the difference between OBP- treated and non-treated HconFs.

Supplementary Figure 3

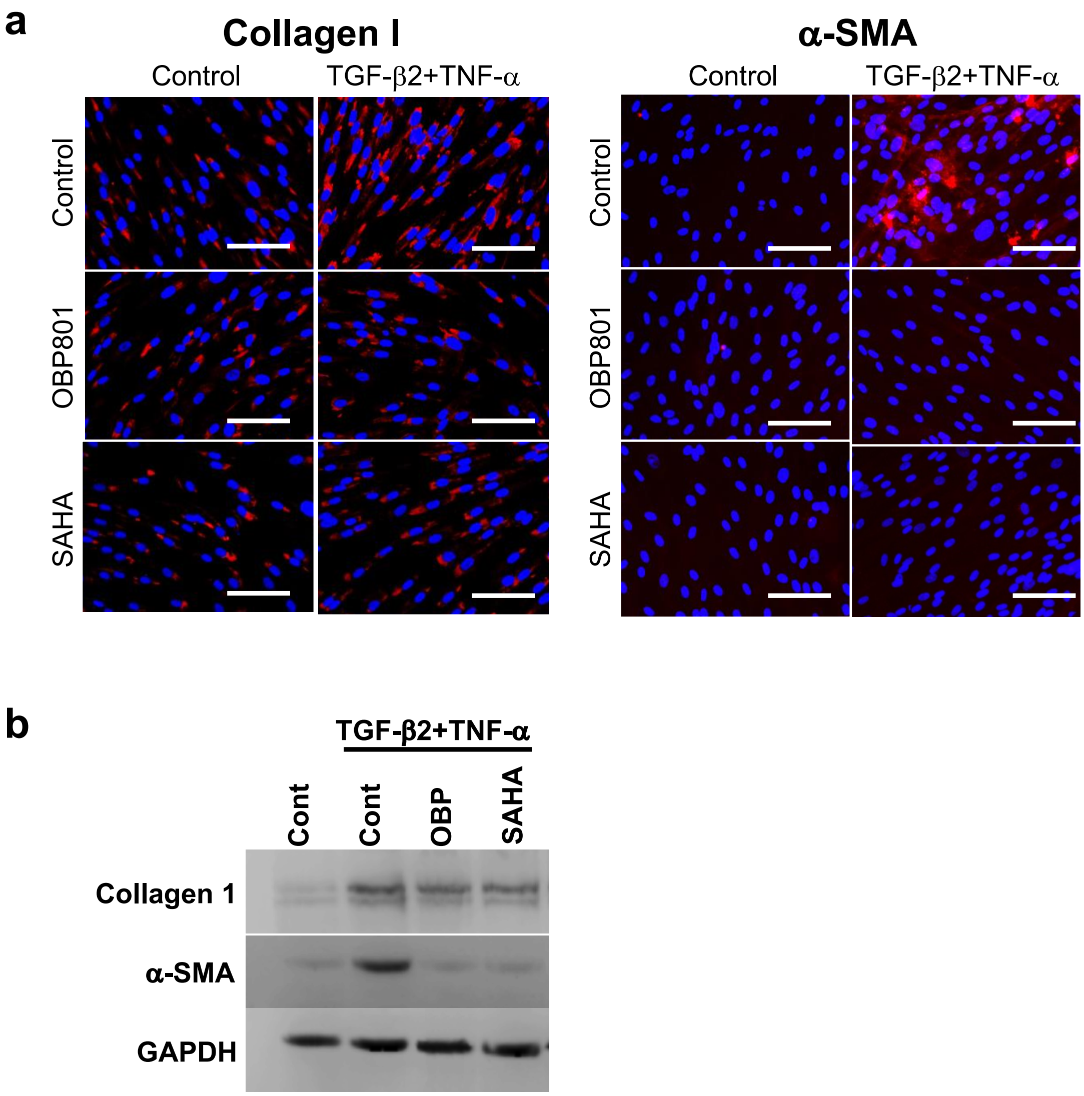

Supplementary Figure 3. Comparison of the effects of OBP and SAHA on expression of α-SMA and collagen type 1. HConFs were pretreated with OBP (1 nM) or SAHA (500 nM) for 24 hours and were stimulated with 20 ng/mL TGF-β2 + 10 ng/mL TNF-α for 48 hours. The samples were evaluated by immunocytochemistry (a) and western blot (b). TGF-β2+TNF-α induced α-SMA and collagen type 1 expression (Red) was reduced by OBP and SAHA pretreatment. Scale bar = 100 μm.

Supplementary Figure 4

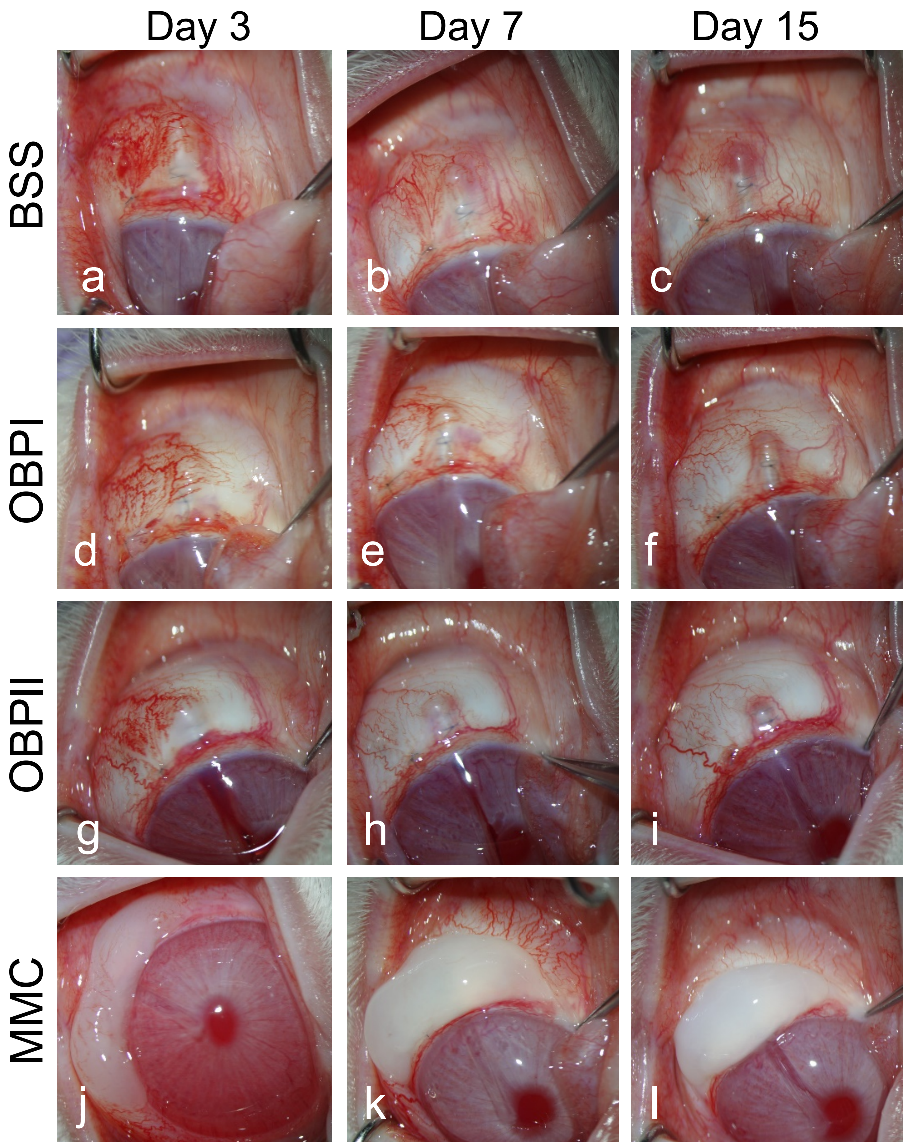

Supplementary Figure 4. Macroscopic postoperative photographs of rabbit eyes at day 3, 7, and 15 after surgery. (a-c) Treatment with BSS was associated with a flat bleb with hyperemia. (d-i) OBP treated eye showed a flat, functional bleb with decrement of hyperemia. (j-l) Treatment with MMC was associated with an avascular, thin, and cystic bleb, and the conjunctiva around the bleb was scarred, thus resulting in an encapsulated bleb.

Supplementary Figure 5

Western blot for Fig. 3

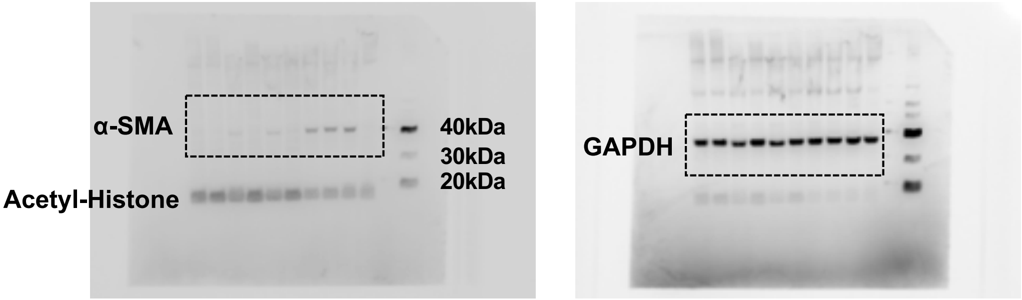

Western blot for Fig. 5

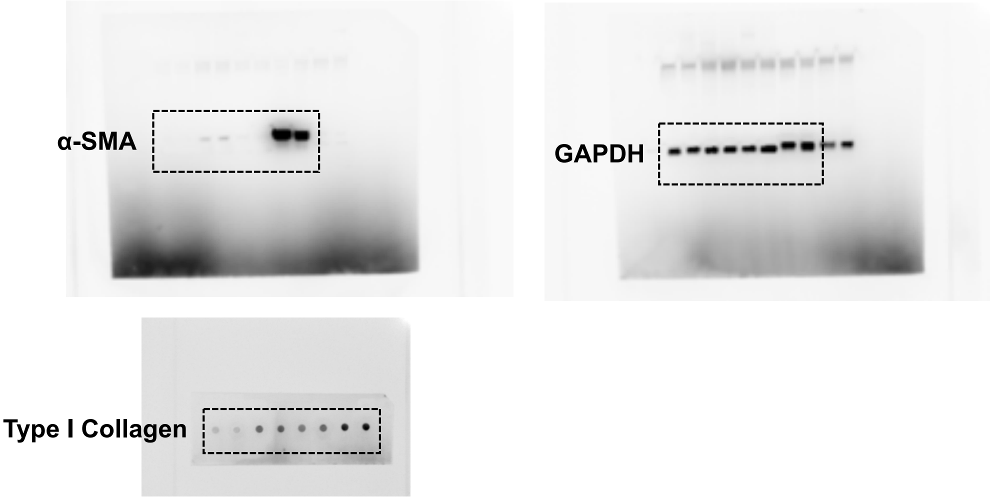

Western blot for Supplementary Fig. 3b

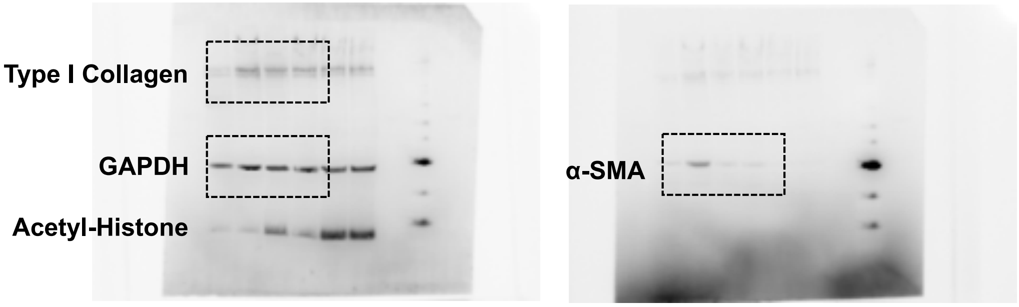

Supplementary Figure 5. The original images of full-length blots of the indicated figures. Dashed squares indicate areas used for the figures.

Supplementary Table 1. List of fibrosis associated genes analyzed in this study

Growth Factors	CTGF, PDGFA, PDGFB, VEGFA
Extracellular Matrix Components	COL1A2, COL3A1, COL4A2, COL16A
Remodeling Enzymes	LOX, LOXL1, LOXL2, LOXL3, LOXL4, MMP2, MMP9, MMP14 PLAT (tPA), PLAU (uPA), SERPINB2, SERPINE1 (PAI-1), SERPINH1, TIMP1, TIMP2, TIMP3,
Cellular Signaling	TGFβ2, TGFβ3, THBS1
Epithelial-to-Mesenchymal Transition	COL1A2, COL3A1, MMP2, MMP9, MMP2, MMP9, SERPINE1, TGFβ2, TGFβ3
Myofibroblast Activation	ACTA2

Supplementary Table 2. List of primers for qRT-PCR analysis

Primers	Catalog Number	Gene Name
Hs01028956_m1	4453320	COL1A2
Hs00943809_m1	4453320	COL3A1
Hs01098873_m1	4453320	COL4A2
Hs01083497_m1	4448892	COL16A
Hs00942480_m1	4453320	LOX
Hs01046941_g1	4448892	LOXl3
Hs00260059_m1	4453320	LOXl4
Hs00158757_m1	4453320	LOXl2
Hs00935937_m1	4453320	LOXl1

Expanded Material and Methods

Reverse transcriptase-polymerase chain reaction (RT-PCR) analysis

In quantitative (q)RT- PCR assays, complementary DNA (cDNA) was synthesized using the High-Capacity cDNA Reverse Transcription kit (Thermo Fisher Scientific). PCR reactions used the TaqMan Fast Advanced Master Mix (Thermo Fisher Scientific) with TaqMan primers (Thermo Fisher Scientific) (Supplementary Table 1). In RT-PCR arrays, cDNA was synthesized using the RT2 First Strand kit (QIAGEN). Thermal cycling as recommended by the manufacturer for ABI StepOnePlus (Applied Biosystems) was used with RT2 Profiler PCR Array Human Fibrosis (QIAGEN). Data Analysis v3.5 (QIAGEN) software was used.

Western blot analysis

The blocking and detection reaction were performed using iBind Western System (Thermo Fisher Scientific), after SDS-PAGE electrophoresis on 4–12% gel. The primary antibodies, mouse anti-actin α-smooth muscle (α-SMA) (1:1,000) (Sigma-Aldrich, St. Louis, MO, USA) and anti-Collagen (1:500) (Abcam, Cambridge, MA, USA) antibodies, were used, and goat-anti-mouse and rabbit IgG HRP conjugate (1:1,000) (Southern Biotech, Birmingham, AL, USA) antibodies were used as secondary antibodies. The protein bands were made visible with an ECL chemiluminescent substrate reagent kit (Thermo Fisher Scientific). Luminescence was observed with ImageQuant LAS-3000 (FUJIFILM, Tokyo, Japan), a dedicated charge-coupled device (CCD) camera system. Semi-quantification of the density of each band was conducted using the Image J software program (1.47v; NIH, Bethesda, MD, USA) with GAPDH as the reference protein.
